# Supplementary figures and images for: Depth and benthic habitat influence shallow and mesophotic predatory fishes on a remote, high-latitude coral reef
Source: PLoS One. 2022 Mar 24;17(3):e0265067. doi: 10.1371/journal.pone.0265067 (PMC8947262; doi:10.1371/journal.pone.0265067)

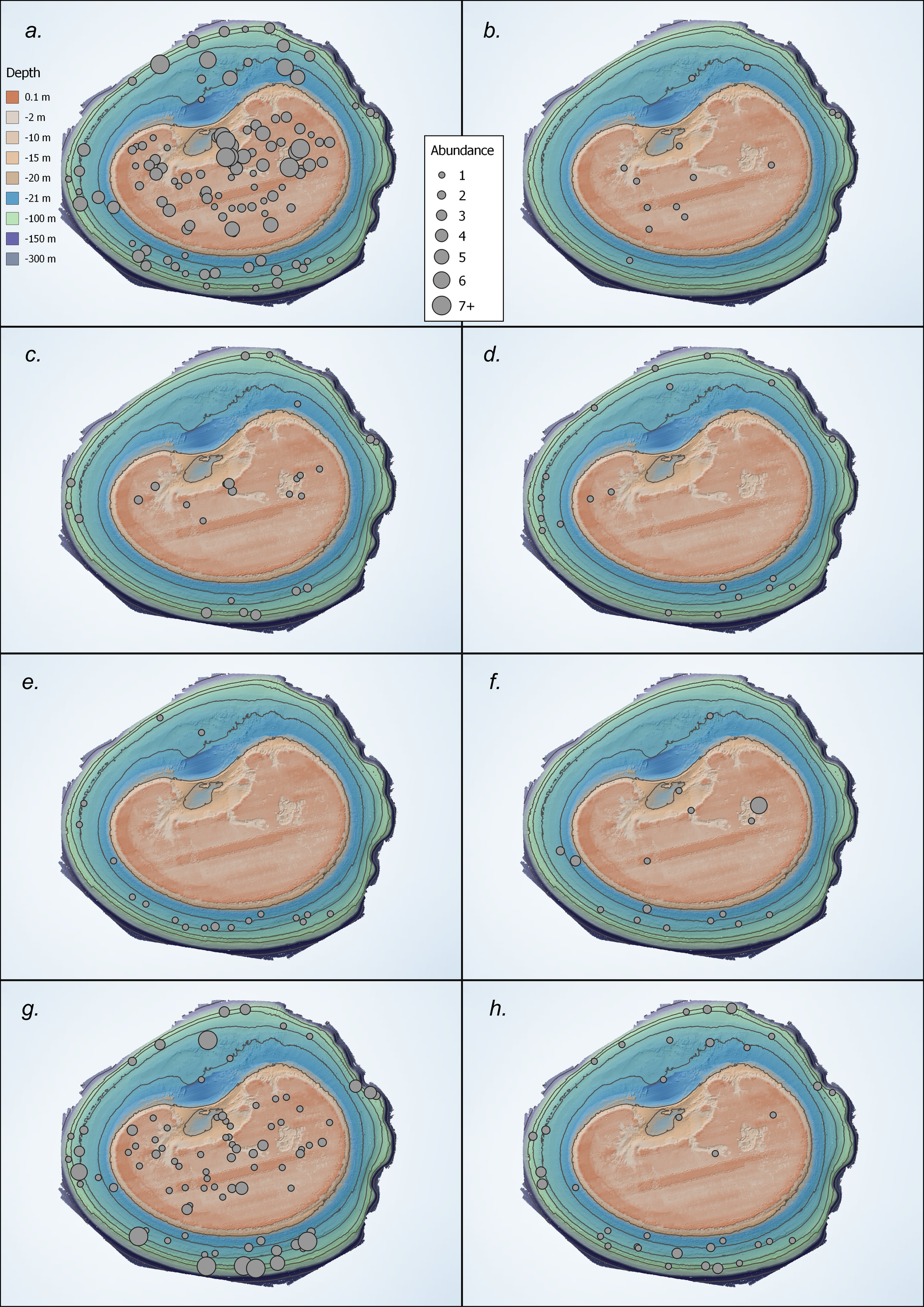

Supplement: S1 Fig — a. Carcharhinus galapagensis, b. Galeocerdo cuvier, c. Epinephelus daemelii, d. E. cyanopodus, e. Lethrinus rubrioperculatus, f. Lutjanus bohar, g. Seriola lalandi, h. S. rivoliana, i. Pristipomoides filamentosus, j. Aprion virescens. (TIF) [file pone.0265067.s001.tif]

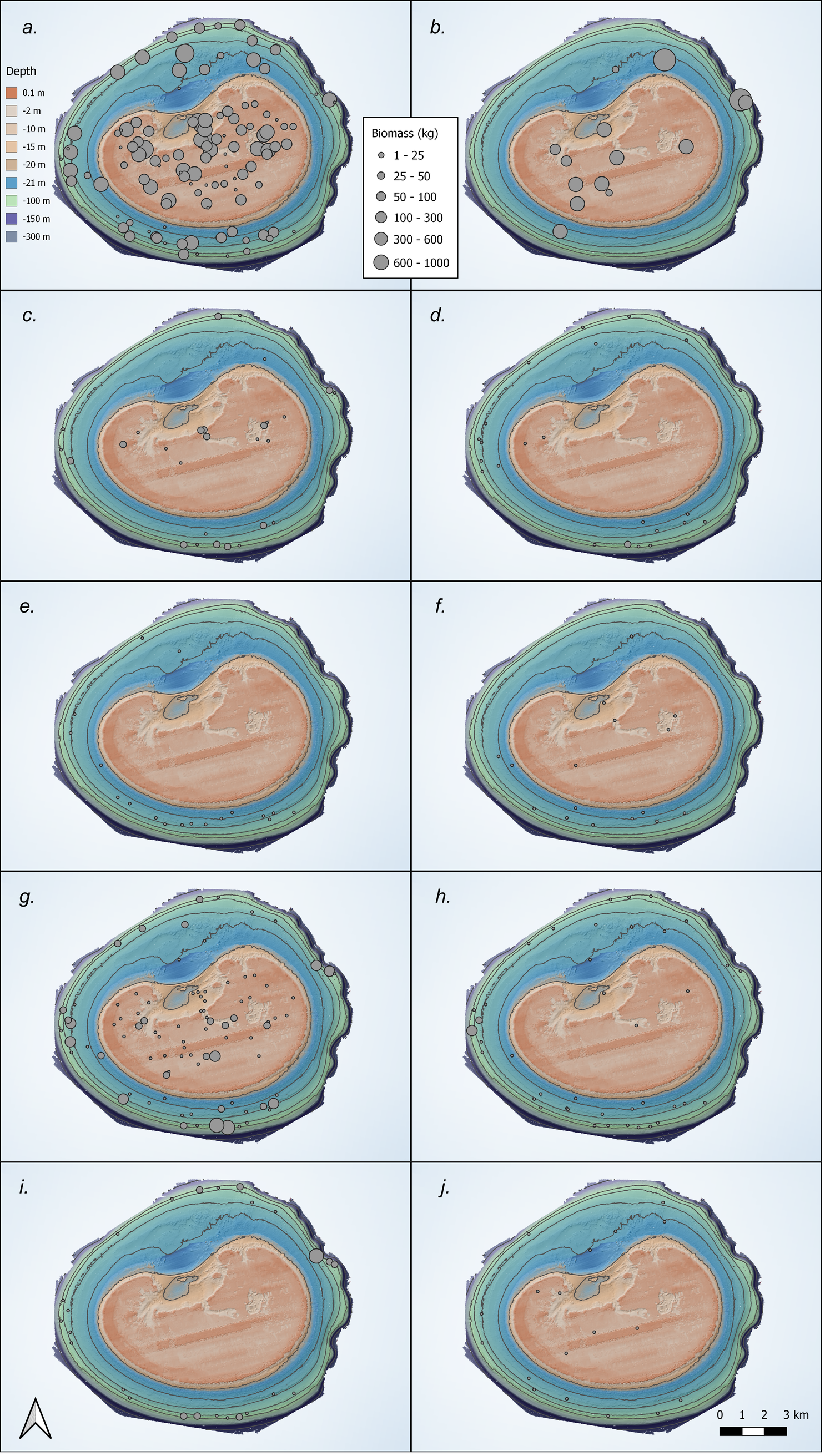

Supplement: S2 Fig — a. C. galapagensis, b. G. cuvier, c. E. daemelii, d. E. cyanopodus, e. L. rubrioperculatus, f. L. bohar, g. S. lalandi, h. S. rivoliana, i. P. filamentosus, j. A. virescens. (TIF) [file pone.0265067.s002.tif]
