# Supplementary material for: Depth and benthic habitat influence shallow and mesophotic predatory fishes on a remote, high-latitude coral reef
Source: PLoS One. 2022 Mar 24;17(3):e0265067. doi: 10.1371/journal.pone.0265067 (PMC8947262; doi:10.1371/journal.pone.0265067)
Supplement: S1 Table — Abundance based on summed MaxN; standardized abundance based on number of deployments per area (lagoon 71, north 25, south 35) and calculated total biomass. (DOCX) [file pone.0265067.s003.docx]

**S1 Table.** Predatory fishes recorded in stereo-BRUV deployments on Middleton Reef. Abundance based on summed MaxN; standardized abundance based on number of deployments per area (lagoon 71, north 25, south 35) and calculated total biomass.

| **Family** | **Scientific Name** | **Common Name** | **Abundance** | **Standardized abundance (abundance/deployments)** | | | **Biomass (kg)** | **Trophic Guild (RLS; *Fishbase, ** genus guild)** | **Predatory group** | **IUCN Listing Status** | **Fisheries Mortality Australia** |
| --- | --- | --- | --- | --- | --- | --- | --- | --- | --- | --- | --- |
|  |  |  |  | **Lagoon** | **North** | **South** |  |  |  |  |  |
| *Aulostomidae* | *Aulostomus chinensis* | Trumpetfish | 7 | 0.06 | 0.12 |  | 128 | generalist carnivore | Meso | least concern | N |
| *Belonidae* | *Tylosurus crocodilus* | Crocodile longtom | 8 | 0.11 |  |  |  | invertebrate carnivore | Meso | least concern | Y |
| *Bothidae* | *Bothus pantherinus* | Leopard flounder | 1 | 0.01 |  |  |  | *invertebrate carnivore | Meso | least concern | Y |
| *Bothidae* | *Bothus sp* | Flounder | 1 |  | 0.04 |  |  | *invertebrate carnivore | Meso |  |  |
| *Carangidae* | *Carangoides orthogrammus* | Thicklip trevally | 21 | 0.25 |  | 0.09 | 41 | generalist carnivore | Meso | least concern | Y |
| *Carangidae* | *Caranx lugubris* | Black trevally | 2 |  |  | 0.06 | 7 | piscivore | Meso | least concern | Y |
| *Carangidae* | *Seriola lalandi* | Yellowtail kingfish | 186 | 0.85 | 1.6 | 2.46 | 2202 | generalist carnivore | Apex | least concern | Y |
| *Carangidae* | *Seriola rivoliana* | Highfin amberjack | 69 | 0.03 | 0.72 | 1.4 | 357 | piscivore/generalist carnivore | Meso | least concern | Y |
| *Carcharhinidae* | *Carcharhinus galapagensis* | Galapagos shark | 329 | 2.54 | 3.32 | 1.89 | 9615 | *generalist carnivore | Apex | least concern | Y |
| *Carcharhinidae* | *Carcharhinus plumbeus* | Sandbar shark | 1 |  | 0.04 |  | 18 | generalist carnivore | Apex | vulnerable | Y |
| *Carcharhinidae* | *Galeocerdo cuvier* | Tiger shark | 14 | 0.11 | 0.2 | 0.03 | 3096 | generalist carnivore | Apex | near threatened | Y |
| *Dasyatidae* | *Dasyatis thetidis* | Black stingray | 17 | 0.17 | 0.12 | 0.06 | 245 | generalist carnivore | Meso | least concern | Y |
| *Dasyatidae* | *Taeniurops meyeni* | Blotched fantail ray | 7 | 0.01 | 0.08 | 0.11 | 30 | generalist carnivore | Meso | vulnerable | Y |
| *Fistulariidae* | *Fistularia commersonii* | Smooth flutemouth | 7 | 0.07 | 0.04 | 0.03 | 13 | generalist carnivore | Meso | least concern | N |
| *Grammistidae* | *Aulacocephalus temminckii* | Goldribbon cod | 2 |  | 0.08 |  |  | *generalist carnivore | Meso | least concern | N |
| *Lethrinidae* | *Lethrinus miniatus* | Redthroat emperor | 10 |  |  | 0.29 | 41 | generalist carnivore | Meso | least concern | Y |
| *Lethrinidae* | *Lethrinus rubrioperculatus* | Spotcheek emperor | 19 |  | 0.08 | 0.49 | 23 | invertebrate carnivore | Meso | least concern | Y |
| *Lutjanidae* | *Aprion virescens* | Green jobfish | 19 | 0.07 | 0.2 | 0.26 | 137 | generalist carnivore | Meso | least concern | Y |
| *Lutjanidae* | *Lutjanus bohar* | Red bass | 25 | 0.13 | 0.08 | 0.4 | 103 | generalist carnivore | Meso | least concern | Y |
| *Lutjanidae* | *Pristipomoides filamentosus* | Rosy snapper | 137 |  | 3.68 | 1.29 | 606 | *generalist carnivore | Meso | least concern | Y |
| *Muraenidae* | *Gymnothorax annasona* | Lord Howe moray | 14 | 0.06 | 0.04 | 0.26 | 5 | **generalist carnivore | Meso | least concern | N |
| *Muraenidae* | *Gymnothorax meleagris* | Whitemouth moray | 1 | 0.01 |  |  |  | *generalist carnivore | Meso | least concern | N |
| *Muraenidae* | *Gymnothorax nubilus* | Grey moray | 1 | 0.01 |  |  |  | **generalist carnivore | Meso | least concern | N |
| *Muraenidae* | *Gymnothorax thyrsoideus* | Greyface moray | 8 | 0.11 |  |  |  | invertebrate carnivore | Meso | least concern | N |
| *Serranidae* | *Acanthistius cinctus* | Yellowbanded wirrah | 2 | 0.01 | 0.04 |  |  | *generalist carnivore | Meso | least concern | N |
| *Serranidae* | *Cephalopholis argus* | Peacock rockcod | 6 |  | 0.04 | 0.14 | 10 | piscivore | Meso | least concern | Y |
| *Serranidae* | *Cephalopholis miniata* | Coral rockcod | 2 |  |  | 0.06 | 1 | piscivore | Meso | least concern | Y |
| *Serranidae* | *Epinephelus cyanopodus* | Purple rockcod | 20 | 0.03 | 0.24 | 0.34 | 211 | *generalist carnivore | Meso | least concern | Y |
| *Serranidae* | *Epinephelus daemelii* | Black rockcod | 42 | 0.24 | 0.28 | 0.51 | 653 | generalist carnivore | Apex | near threatened | Y |
| *Serranidae* | *Epinephelus maculatus* | Highfin grouper | 19 | 0.07 | 0.36 | 0.14 | 28 | generalist carnivore | Meso | least concern | Y |
| *Serranidae* | *Epinephelus morrhua* | Comet grouper | 10 |  | 0.12 | 0.2 | 37 | *generalist carnivore | Meso | least concern | Y |
| *Serranidae* | *Epinephelus rivulatus* | Chinaman rockcod | 15 | 0.01 | 0.16 | 0.29 | 10 | generalist carnivore | Meso | least concern | Y |
| *Serranidae* | *Epinephelus tauvina* | Greasy rockcod | 1 |  |  | 0.03 | 3 | piscivore | Meso | data deficient | Y |
| *Serranidae* | *Trachypoma macracanthus* | Pacific rockcod | 1 | 0.01 |  |  |  | *generalist carnivore | Meso | least concern | N |
| *Serranidae* | *Variola louti* | Yellowedge coronation trout | 10 | 0.04 | 0.08 | 0.14 | 75 | piscivore | Meso | least concern | Y |
| *Tetraodontidae* | *Lagocephalus sceleratus* | Silver toadfish | 10 |  | 0.36 | 0.03 | 106 | generalist carnivore | Meso | least concern | Y |
| **Total** | |  | 1044 | 5.03 | 12.12 | 10.97 | 17803 |  |  |  |  |
