# Supplementary material for: Depth and benthic habitat influence shallow and mesophotic predatory fishes on a remote, high-latitude coral reef
Source: PLoS One. 2022 Mar 24;17(3):e0265067. doi: 10.1371/journal.pone.0265067 (PMC8947262; doi:10.1371/journal.pone.0265067)
Supplement: Attachment — Submitted filename: ReviewPlosOne_Brown_BRUVS.docx [file pone.0265067.s004.docx]

Review for PLOS One

Depth and benthic habitat influence shallow and mesophotic predatory fishes on a remote, high-latitude coral reef

Brown, Monk, Williams, Carroll, Harasti, Barrett

Spatial distribution and extent of predatory fish in shallow to mesophotic shelf environments

BRUVS lagoon and outer shelf habitats 0-100 m

Remote location subject to varied oceanographic processes

FYI, Check journal’s taxon naming specifications before publication, for JFB the common name and authority/year need to be included at first mention of species. While some common names are included not all species in the text have common names mentioned, also be careful of capitalisation depending on if you are using common names or the Australian Standard Names (e.g. Fishes of Australia website for correct info). Would be helpful to include more common names as it could help internet searches to your publication and also is helpful to include in tables, etc for enhanced understanding.

Abstract:

Line 20: Add “fish” inbetween “predatory populations”

Line 27: “…five predatory fish” makes it sound like you will list them. Not necessary here because of abstract word limits but I would suggest replacing “while” with “and” and starting a new sentence about Galapagos shark and Black cod or in some way splitting that long sentence.

Introduction:

Line 28: be consistent with capital letters and if you ae using Australian Standard Names (both capitalised) or ‘common names’ are generally not capitalised. Fishes of Australia website has the correct ASN

Line 47: Baseline understanding (singular?)… is necessary

Line 60-62: split the sentence/re-write

Materials and methods

Line 92: oceanographic influences (plural?)

Line 93: replace influence with strength or magnitude?

Line 101: add latitudinal degree and specify typical of tropical or shallow-water corals (vs deepwater coral latitudinal limits)

Line 105: “The reef structure morphology resembles a system that is modified by both hard coral formation and rubble accretion, which reflects seasonal variation…” This sentence could be improved.

Line 119: “across depths from just below the surface to 100 m deep.”

Line 121: Source of existing bathymetry? Geoscience Australia or?

Line 125: austral summer

Paragraph beginning Line 118 or Line 123: add approx minimum space between deployments to maintain independence of replicates (on the map they look close, but that is probably just the size of the markers)

Line 130: Sardinops sagax

Line 138: position of “.” Should be “GoPro .mp4”?

Line 140: “within an estimated 8 m field of view”

Line 142: “and length measurements were taken for each individual encountered”

Line 143: “For teleost fishes, length was measured from the most anterior position (snout) to tail fork. Rays were measured across the widest portion of the disc. Other elasmobranchs were measured from snout to posterior tip on caudal fin.” Was this total length?

Line 145: “When fish were in large schools…” here you are referring to a school of one species or a school with more than one species? I would re-write this sentence to specify that you measured 20 individuals of the same species.

Line 154: either a comma is needed between the common and scientific names or move the parentheses?

Paragraph beginning Line 157: more information is needed like the number of habitat points per deployment or was this based on estimated percent cover? How many habitat categories and what were they? From what I can remember CATAMI just specified the naming convention/types/categories, but not specifically how to measure/quantify within a BRUVS field of view.

Line 163” “data did not need to be transformed”? Not sure if untransformed is a word. Also, raw data does not necessarily need to be transformed but I would clarify if you used any tests (visual or statistical) to check the distribution of data, etc? Also, if this is the first mention of Primer, please include the software details here.

Line 164: One outlier… I would add more information here. Was this a replicate where no individuals were sampled?

Line 168: “across the fixed factor area”? Also this sentence is confusing how it is worded, perhaps move the clause “on the predator…and biomass structure” to the end of the sentence. Also, in this instance can “predator community assemblage abundance” be shortened to “predator relative abundance”?

Line 169: I would clarify that the SIMPER function is the “percentage similarity of….” And clarify if you used presence-absence or abundance data.

Line 173: missing “transformed”

Line 174: “Vectors representing the most influential species/habitat variables were overlaid…”?

Paragraph beginning Line 176 could be re-written for clarity with more details. E.g., “To understand the relationships between taxa and measured habitat variables, a number of analyses were conducted. Species influential in distinguishing communities among areas around Middleton Reef (north, south, lagoon) from the SIMPER analysis were then used to test the relationships between habitat variables. Only species with sufficient abundance (MaxN > xx or n ≥ xx ) were included.”

Line 182: split up sentence for clarity and include a citation for Tweedie distribution being an appropriate choice.

Line 184-185: Change “3” to “three”

Line 192: here the use of ‘predatory’ seems like the species accumulation curve was being aggressive. Perhaps change to Predator species accumulation curve or Species accumulation curves of predatory species? Or add a ‘ after species. Line 202 and 204 same comment. Note this is just a suggestion but I think predator species sounds better than predatory species also throughout the results section.

Line 215: This is not the correct common name for *Pristipomoides filamentosus*. It is easy to be confused because there are many names but most often Rosy Jobfish (ASN) or crimson jobfish are used, sometimes rosy snapper. This is to differentiate it from the *other* deepwater and eteline snappers (e.g. lavendar jobfish, ruby snapper, flame snapper and the half dozen species referred to as ‘red snapper’) ;-)

Results

The results section is well-written. I only have a few comments.

Line 263 I think Pearson should be capitalised.

Table 3: Not necessary but it might be nice to have some way of distinguishing the most similar/dissimilar, either with color, font differences (i.e. bold) or like a heat map.

Line 302: Remove the apostrophe after GAM, should be GAMs

Line 317: In serranid the ‘s’ should be lowercase but for Serranidae the ‘s’ is capitalised.

Table 5: Great table!

Discussion

Overall I found the Discussion well-written and interesting. I only have a few comments.

Paragraph beginning Line 339: This is a big paragraph and might benefit from splitting. Perhaps around “Complex habitat…” how you do it is up to you.

Line 372: change fishing to “fishing effort”

Line 381: This first/topic sentence lacks a little oomph. I think it can be fixed by switching the order of words or a different word than concide. It does not match with the direction of the paragraph.

Line 391: I would write out East Australian Current (EAC) since it is the first mention in the Discussion.

Conclusion

Line 402-403: This sentence could be re-worded/re-structured for clarity

Line 407: beter segue between sentences? Or the sentence beginning with “Declines…” could be moved to the beginning of the paragraph
